# Novel Integrated Flow-Based Steam Distillation and Titration System for Determination of Volatile Acidity in Wines

**DOI:** 10.3390/molecules26247673

**Published:** 2021-12-18

**Authors:** Justyna Paluch, Joanna Kozak, Karolina Mermer, Iwona Molęda, Marcin Wieczorek, Sławomir Kalinowski, Paweł Kościelniak

**Affiliations:** 1 Faculty of Chemistry, Jagiellonian University, Gronostajowa 2, 30-387 Krakow, Poland; karolina.mermer@doctoral.uj.edu.pl (K.M.); iwonalo@interia.pl (I.M.); marcin.wieczorek@uj.edu.pl (M.W.); pawel.koscielniak@uj.edu.pl (P.K.); 2 Department of Chemistry, University of Warmia and Mazury, Plac Łódzki 4, 10-957 Olsztyn, Poland; kalinow@uwm.edu.pl

**Keywords:** spectrophotometry, automated steam distillation, monosegmented flow titration, flow analysis, wine analysis

## Abstract

Novel integrated flow-based steam distillation and titration system with spectrophotometric detection was developed for determination of volatile acidity in wines. Using the system, the distillation procedure was carried out in an automatic manner, starting with introducing into a heated steam distillation module a sample and subjecting it to steam distillation. Under selected conditions, all the analyte was transferred to the distillate; therefore, the system did not require calibration. The collected distillate and titrant were introduced into the next monosegments in varying proportions, in accordance with the developed titration procedure, and directed to the detection system to record the titration curve. The titration was stopped after reaching the end point of titration. Procedures for distillation and titration were developed and verified separately by distillation of acetic acid, acetic acid in the presence of tartaric acid as well as acetic acid, tartaric acid, and titratable acidity, with precision (relative standard deviation) and accuracy (relative error) for both procedures lower than 6.9 and 5.6%, respectively. The developed steam distillation and titration systems were used to determine volatile acidity in samples of white and rosé wines separately and as the integrated steam distillation and titration system, both with precision lower than 9.4% and accuracy better than 6.7%.

## 1. Introduction

Volatile acidity (VA) is one of the most important parameters determined during production and storage of wine. It is important due to chemical and microbiological stability of wine, preserving its specific taste and color, and it is linked to wine quality [1]. The VA value corresponds to the acids that can be removed by steam distillation, and it is influenced by the content of volatile carboxylic acids, mainly acetic acid, but also formic, butyric, and propionic acids [1,2,3]. These acids are present in all wines, but only in microbiologically spoiled wines at levels that are sensorially detectable [3]. The VA is usually expressed as the content of acetic acid [2,4]. Acetic acid is formed during and after alcoholic fermentation [2,3]. Its content in wine usually ranges from 0.4 to 0.8 g L^−1^ and does not exceed 1.1 g L^−1^ [1]. The maximum level of acetic acid, fixed in the European Community by the International Organization of Vine and Wine (OIV), is 1.2 g L^−1^ [5]. Volatile acids are separated from wine by steam distillation and titrated using standard sodium hydroxide [4]. Volatile acids and poorly volatile acids corresponding to the fixed acidity, such as, inter alia tartaric, malic, citric, and lactic acids making up the structure of the wine, constitute the titratable (total) wine acidity [1]. It is expressed as the tartaric acid content and determined by titrating a given volume of sample with sodium hydroxide using phenolphthalein as an acid–base indicator [1,4,6].

Methods of flow analysis (inter alia, Flow Injection Analysis, FIA, Sequential Injection Analysis, SIA, or Multicommuted Flow Analysis, MCFA) are often used to improve as well as to develop novel analytical methods [7,8,9,10]. Their advantages include the possibility of mechanization or automation of all analytical processes, increasing work safety, high sample throughput, and reducing consumption of reagents and sample, and, consequently, waste production [10,11,12]. As a result, they fit into the rules of Green Analytical Chemistry [13,14].

Many flow-based methods were developed to determine titratable acidity in wines [15,16,17,18,19,20,21,22,23,24,25,26,27,28]. However, only a few of them were simultaneously adapted to VA determination [21,22,23,24,25,26,27,28]. The procedure of VA determination is laborious and time-consuming. It includes two stages: the separation of volatile fraction and the VA determination. To improve and automate these processes, membrane-based methods utilizing pervaporation (PV) [22,23,24,25] or gas-diffusion (GD) [21,26,27,28] modules were implemented using FIA [21,22,23,24,27] or SIA [25,26] systems. The systems employed electrophoretic separation [22] and spectrophotometric [21,22,23,24,25,26], conductometric [21] detection, bulk acoustic wave-impedance sensor [27], or pH-ISFET (Ion-Sensitive Field Effect Transistor) [28].

When using PV or GD systems, typically, an aspirated sample was propelled toward the PV module [25] or the donor channel of the GD module [26]. In the established conditions, volatile acids were transferred to the acceptor solution containing the bromothymol blue (BTB). The reaction product formed in an acceptor solution was transported to the spectrophotometric detection system, where the change of the color of BTB in the presence of the acidity compounds was monitored [25,26]. Using the PV systems, the VA was determined in the range of 0.1–0.9 g L^−1^ [22,23,24] or 0.1–1.5 and 1.5–6.0 g L^−1^ [25,29] with precision reported only in two methods (relative standard deviation, RSD) at 25% [22] and 5% [25]. Using the GD systems, the VA was determined generally up to 0.6 [27] or 1.1 g L^−1^ [21,26] with precision lower than 1.0 [21] or 5.1% [26]. The advantage of using membrane-based systems is minimum sample pretreatment. However, they may be prone to membrane contamination and calibration is necessary to determine the VA.

Considering the features of the systems described above, the aim of this work was to develop a flow-based system allowing for automation of the steam distillation process, recommended for the VA determination [6], and integrating both steam distillation and titration procedures. To automate the steam distillation procedure, an SIA system with a preconcentration module operating in the flow mode [29] was adapted. The system was initially developed to preconcentrate the sample in a continuous mode based on membraneless evaporation under reduced pressure. The evaporation module consisted of a cylinder glass tube (of 15 mL capacity) located inside an aluminum block used for its thermostating. The tube was covered at the top with a (polytetrafluoroethylene) PTFE block with an inlet (i.d. of 0.8 mm) for sample dispensation. The upper part also included connections with a vacuum pump and a pressure gauge. The lower part of the cylinder was also closed by PTFE block with a narrow outlet (i.d. of 0.8 mm) allowing even a small amount of preconcentrated sample to be collected and introduced into the flow system. During the evaporation process, the dosed drops of solution fell freely to the bottom of the vessel. In the research presented in this paper, the module was adapted to perform the steam distillation process.

Using the developed systems, titratable acidity was determined in a flow-based titration mode [15,16,17,18,19,20]. Flow-based techniques offer a wide range of possibilities to automate titration. Using the flow systems, it can be carried out in accordance with the conventional procedure. However, numerous novel titration modes were developed. They can be divided into titration based on flow of stream or segment of sample, sample monosegment formation, or a combination of different flow-based approaches [30]. MCFA systems were developed for determination of titratable acidity using titration based on the conventional mode [16] or titration based on merging sample and titrant aliquots, following the binary search concept, in which the course of titration is decided based on previous measurements [17]. The FIA system for titration based on forming concentration gradients and using a single standard solution to calibrate the gradient was developed [15] as well as several systems for so-called FIA titration requiring calibration using a set of standards solutions [18,19,20].

Regarding monosegmented flow titration, it is based on formation of segments of a mixture of sample and titrant separated on both sides from the carrier by segments of air (or inert gas) [31,32,33,34,35,36,37,38,39]. Sample and titrant can be introduced into monosegments in various ways. In the system described in [35], a portion of titrant was dosed by a burette to a monosegment containing a sample. The content of the monosegment was mixed and directed to the detector. In a different approach to titration, the volumes of the titrant introduced into the monosegment [31,36] or the volumes of both the titrant and the sample [33,34] were changed. The endpoint was determined based on titration curves representing the relation between signal and titrant volume [33] or time [36], or using an appropriate numerical algorithm [31,34]. Monosegmented titration was also carried out using Lab-on-Valve [37] or sequential injection [38] systems in which segments of air, sample, indicator, titrant, and then air were introduced into the holding coil in turn. Calibration using a set of standard solutions was necessary to perform the determination.

The titration proposed in this work is the first adaptation to acid–base titration, of the method developed for complexometric Fe(III) titration with spectrophotometric detection [39]. It consisted in creating in the flow system different, precisely defined concentration gradients of titrant and analyte in each of the successively formed monosegments and was based on using the calculated titrant dilution factor. The reason for using this titration system was its automation and the possibility of integrating with the part of the flow system containing the steam distillation module, of determining the analyte in a wide concentration range without the necessity of system or method calibration, and low sample consumption (4 mL).

In this work, an original automated flow system was developed to integrate steam distillation and titration (SD–T) processes for determination of volatile acidity in wines. Using the established instrumental conditions, a volume of distillate, corresponding to the distillation of the whole amount of analyte, was collected, so there was no need to calibrate the system. Then, the distillate fraction was titrated using the on-line titration system. Procedures for distillation and titration were developed and verified by distillation of acetic acid, acetic acid in the presence of tartaric acid as well as determination of acetic acid, tartaric acid, and titratable acidity. Finally, the SD–T system was applied to analyze the white wine samples.

## 2. Results

### 2.1. Integrated Flow System for Automated Steam Distillation and Titration

**Figure 1:**
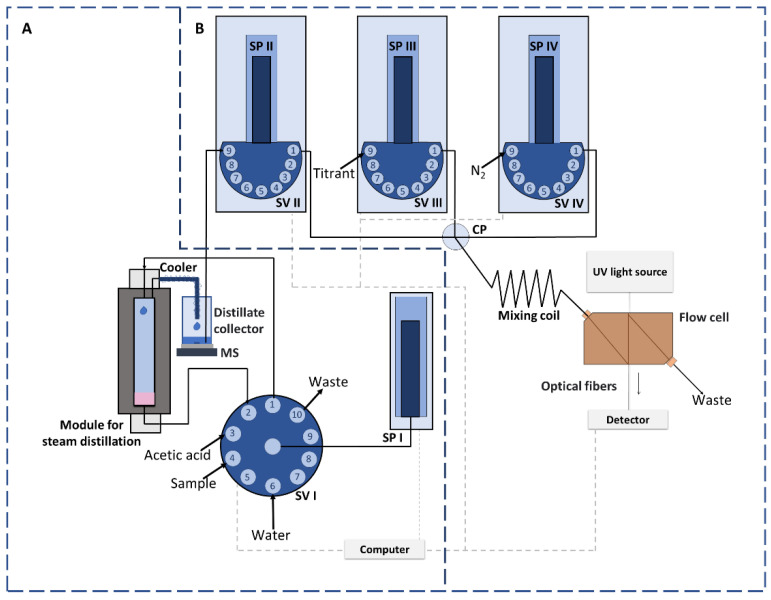
Integrated flow system designed for steam distillation (part **A**) and titration (part **B**). SP I–IV—syringe pumps, SV I–IV—selection valves, CP—confluence point, MS—magnetic stirrer.

An integrated flow system was proposed to perform on-line steam distillation and titration processes for determination of volatile acidity in wines. The scheme of the developed steam distillation and titration (SD–T) system is presented in Figure 1. The system consisted of a steam distillation (Figure 1A) and a titration (Figure 1B) part.

The main element of part A of the constructed flow system (Figure 1A) was a steam distillation module (SDM). The same module, with a different configuration, was previously applied as a preconcentration module for sample evaporation [29]. The module consists of an aluminum block in which a cylindrical glass tube (1.4 × 10 cm) is located. To enable thermostating (in the temperature range of 20 to 150 °C), the block was equipped with a heater and a thermocouple connected to a temperature controller. At the top and the bottom, the heating block was closed with polytetrafluoroethylene (PTFE) blocks, with inlet and outlet holes (at the top, i.d. of 0.8 mm) enabling introduction of sample and water (for steam distillation) and collection of the distillate, respectively, and with an outlet hole (at the bottom, i.d. of 0.8 mm) to remove the remaining fluid from the glass tube. The PTFE tubing used to collect the distillate (i.d. of 0.2 mm) was continuously cooled by water flowing through a wound on PTFE tubing (i.d. of 0.8 mm). The distillate was collected in a closed distillate chamber (of 27 mL capacity) placed on a magnetic stirrer (Figure 1A). The system was equipped with a syringe pump (with a capacity of the syringe of 4 mL), enabling introduction of the sample, dosage of water (used for steam distillation), and removal of solutions from the module. The use of a ten-position selection valve allowed for control of the flow direction of solutions between individual elements of the system.

Part B of the developed flow system was designed for on-line titration (Figure 1B). It consisted of three syringe pumps equipped with nine-position selection valves. Pump II and pump III were used for propelling the sample (from the distillate collector) and titrant, respectively. The third pump was used for nitrogen introduction to create separate segments of different sample and titrant volumes. Nitrogen was aspirated into the system from a tube connected to SV IV (Figure 1B) and a balloon filled with the gas previously. Streams of sample and titrant met at the confluence point and merged in the mixing coil to complete the reaction. The product of the reaction was directed continuously to the flow cell and appropriate signals were measured by the detector.

The operation of the system components was controlled from the system computer. The software allowed for registration of peaks and their visualization in real time.

### 2.2. Steam Distillation (SD) Procedure

The procedure of on-line SD started with introducing an established volume of sample into the glass tube of a heating block heated to 120 °C. Then, after waiting 60 s to heat the sample to a temperature of about 100 °C, water was dispensed into the sample using a syringe pump at a flow rate of 4 µL·s^−1^. The use of a low flow rate of water resulted in immediate evaporation of the dosed drops. Water with volatile sample components was condensed in PTFE tubing (surrounded by a cooler filled with water) and collected in a distillate collector placed on a magnetic stirrer. The purpose of mixing was to ensure homogenization of the obtained distillate. The detailed SD procedure, including washing of the system and aspiration of solutions, is presented in Table 1.

#### 2.2.1. Preliminary Studies

**Figure 2:**
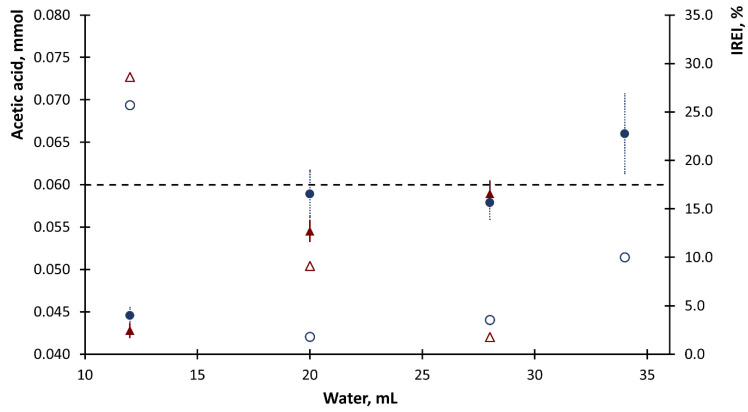
Dependence of the contents of acetic acid in a sample (full mark) and the value of the relative error (empty mark) on the volume of water used for steam distillation for sample volumes of 3 (circle) and 6 (triangle) mL. The values of standard deviation (*n* = 3) are marked with vertical lines and the horizontal line shows the expected value of the number of moles of acetic acid.

The preliminary test included selection of volume of the dosed sample and the volume of water used during the distillation process necessary to transfer the whole amount of analyte into the distillate collector. Acetic acid solutions with concentrations of 20 and 10 mmol·L^−1^ were used as the sample. The use of different concentrations of acetic acid enabled us to maintain a constant absolute number of moles of the acetic acid introduced (in different volumes) into the system at the level of 0.06 mmol. The flow rate of 4 µL·s^−1^ was used for dispensation of water. The tests were performed at two sample volumes: 3 and 6 mL and at four water volumes in the range of 12 to 34 mL. The distillation process was repeated three times. The obtained values of the determined numbers of moles of acetic acid and the calculated relative error (|RE|, %) are shown in Figure 2.

Figure 2 shows that it was necessary to use a min. of 20 mL of water to complete distillation. For this volume, all the acetic acid was transferred to the distillate. However, the water volume of 28 mL was selected for further research because of the better repeatability of the obtained results (*n* = 3). It was found that using 28 mL of water for SD resulted in collecting about 25 mL of distillate, therefore this volume of the sample after distillation was obtained in further studies. These volumes ensured that all the analyte was transferred to the distillate, hence, unlike other methods [21,22,23,24,25,26,27,28], there was no need to calibrate the system. There was no significant difference observed between the results obtained for different sample volumes, but due to the low relative error of the obtained results and better precision, sample volume of 6 mL was selected. However, the possibility of using a sample volume of 3 mL was also confirmed. A smaller volume can be used for samples in which the concentration of acetic acid responsible for volatile acidity is higher. The procedure ensures that the SD process is carried out in a fully mechanized manner, with a time similar to duration of a traditional distillation procedure.

#### 2.2.2. Steam Distillation-Procedure Verification

Procedure of SD was verified through determination of acetic acid in the distillates by traditional titration using NaOH as the titrant and phenolphthalein as the indicator. The procedure was verified by titration of acetic acid in distillates of synthetic samples. The results of the determination of acetic, or acetic and tartaric, acids in synthetic samples contained acetic (Samples 1–5) or a mixture of acetic and tartaric acids (Samples 6–11) at various concentrations as well as values of relative standard deviation and relative errors, as presented in Table 2. The addition of tartaric acid to the synthetic samples made it possible to confirm that tartaric acid had no influence on the obtained acetic acid determination results. The concentration ranges of acetic acid and tartaric acid used correspond to the concentration levels of these acids in wines. Samples were distilled and analyzed three times. It can be concluded that using the developed SD procedure for determination of acetic acid, good values of accuracy (|RE|) and precision (RSD) were obtained, 5.0% and 6.9%, respectively, and no influence of tartaric acid on the determination of acetic acid was observed.

### 2.3. Titration Procedure

To develop an integrated sample distillation and titration system, an on-line distillate titration part of the system was also developed (Figure 1B). To this aim, a previously developed complexometric titration system was adapted for the first time to acid–base titration [39]. To obtain accurate results, it was necessary to reduce the volumes of the sample and the titrant introduced into the monosegments formed in the system to 5 µL, and to develop a novel approach for determination of the end point of the titration.

**Figure 3:**
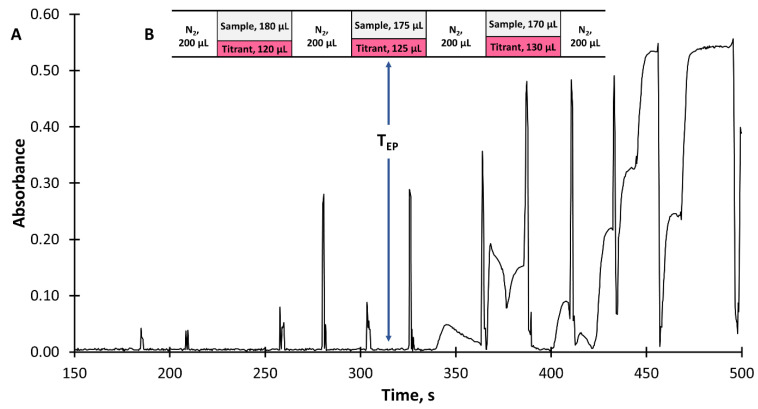
Signals recorded continuously during the on-line titration process (analyte: tartaric acid, 0.17 g L^−1^; titrant: NaOH, 5 mmol L^−1^) (**A**) and monosegments containing sample and titrant mixed with phenolphthalein formed in the system during the titration process (**B**); T_EP_—end point of titration.

The titration procedure consisted of creating monosegments of the same volume in a sequence, containing different but strictly defined volumes of the sample and titrant (mixed with an indicator, phenolphthalein) introduced by syringe pumps SPII and SPIII, respectively (Figure 1B). Nitrogen (200 µL) was introduced using SPIV to form monosegments. The successive monosegments were loaded with smaller (by 5 µL) sample volumes and ever larger (by 5 µL) titrant volumes. To facilitate mixing, the sample and titrant streams were introduced into a monosegment simultaneously, at different flow rates to ensure that appropriate sample and titrant volumes were introduced. The method of introducing the sample and titrant (mixed with phenolphthalein) into the monosegment, and signals recorded during titration, are shown in Figure 3.

In practice, the titration procedure was completed after the end point of the titration was reached. During the research, it was experimentally verified that in the case of the acid–base titration using the proposed mode, the end point of the titration had to be determined based on the volume of titrant corresponding to the monosegment preceding the monosegment for which the signal increase was recorded. This is because the increase in the signal corresponded to an excess of titrant (mixed with phenolphthalein) which remained after the analyte was titrated. The location of the readout of the end point of the titration is shown in Figure 2, and the detailed procedure of titration presented in Table S1 (Supplementary Material). Based on the volumes of sample (V_s_) and titrant (V_Tt_) solutions introduced into the monosegment corresponding to the end point of titration (T_EP_, Figure 2), the titrant dilution factor (f_Tt_), titratable acidity, and VA (C_A,_ g L^−1^, A-analyte) were calculated using Equations (1) and (2), respectively [39]:

f_Tt_ = V_Tt_/(V_Tt_ + V_S_) (1)

C_A_ = (Q·C_Tt_·M_A_·f_Tt_)/(1 − f_Tt_) (2)

where Q is the coefficient resulting from the stoichiometry of the titration reaction (Q = m/n, m-number of moles of analyte, n-number of moles of titrant) and M_A_ is the molar mass of analyte.

#### Titration-Procedure Verification

The flow-based titration procedure was verified through determination of acetic acid (VA), tartaric acid (fixed acidity), and sum of acetic acid and tartaric acid expressed as tartaric acid (titratable acidity). The samples were analyzed three times. The results of titration with values of relative standard deviation and relative errors are presented in Table 3. Calculated values of relative error and relative standard deviation for the results of acetic acid determination were lower than 4.0% and 5.2%, respectively. Using the developed procedure, tartaric acid and titratable acidity were determined with relative error lower than 5.6% and 4.8%, respectively. In all the samples, fixed acidity and titratable acidity were determined with precision (RSD) better than 5.9 and 3.4%, respectively. The results confirmed correct operation of the on-line titration system and the possibility of integrating it with the developed on-line SD system.

### 2.4. Analysis of Real Samples

The developed SD and titration (T) systems were used to determine volatile acidity in samples of dry white and rosé wines separately as well as an automatic integrated SD-T system. Using the integrated SD-–T system, the distillation procedure was carried out in an automatic manner, starting with introducing into the heated SD module 2 mL of water and 1 mL of wine. Then, 28 mL of water was added to the SD module dropwise at 4 mL min^−1^ until 25 mL of distillate was collected. The obtained distillate and titrant were introduced into the monosegments in varying proportions, in accordance with the developed titration procedure, and directed to the detection system to continuously record the titration curve. The titration was stopped after reaching the end point of titration.

Volatile acidity in wine samples, expressed in g L^−1^, was determined directly, and in the same samples spiked with acetic acid (0.60, 0.90, and 1.20 g L^−1^). The recovery method was used to verify the accuracy of the determination of the analyte using the developed method, as the appropriate certified reference material was not available [40].

In cases with the addition of an analyte, the SD procedure presented in Table 1 was modified by skipping steps 9 and 10, and in step 11, instead of 2 mL of water (SV I position 6), acetic acid (SV I, position 3) was introduced and directed to the heating block (step 12). Samples and spiked samples were analyzed three times. The results of the determinations are presented in Table 4. As the recovery method (RV, %) was used to determine the accuracy (and not only to assess the loss of the analyte resulting from the sample processing procedure), in Table 4, the accuracy was expressed in the form of the relative error (RE, %) calculated using the relationship RE = RV − 100. It can be observed that in all the cases the results were obtained with acceptable precision lower than 9.4%, and the precision of the results obtained using SD-–T was slightly better (7.5%) than the precision of the results from using separate SD and T systems. In all the cases, good relative error values, below 6.7%, were obtained. The results may be the basis for a conclusion that the developed, automated, and integrated SD-–T system can be successfully used to determine volatile acidity in wine samples.

## 3. Discussion

The developed novel, automated flow-based system with spectrophotometric detection for determination of VA integrates procedures of steam distillation to separate volatile acids and titration for their determination. Comparing with other methods reported in the literature, they usually use pervaporation or gas diffusion for separating volatile acids [21,22,23,24,25,26,27,28].

The procedure of SD was developed using a module previously developed for sample evaporation under reduced pressure [29]. Using the developed system, 3 or 6 mL of sample was subjected to SD. However, it is possible to use different sample volumes. Using the developed system, the conditions of SD were set in such a way that the entire analyte was transferred to the distillate. Therefore, the advantage of the system is that, unlike the methods presented earlier in the literature [21,22,23,24,25,26,27,28], it does not require calibration.

For titration of the distillate fraction, a titration method based on the formation of monosegments containing different, strictly defined proportions of the sample and titrant was developed. This is the first adaptation to acid–base titration of the previously developed method for complexometric determination of Fe(III) [39]. For this purpose, the volumes of solutions introduced into the monosegments were decreased and the method of determining the end point of titration was developed experimentally. The proposed titration procedure does not require calibration and, depending on the titrant concentration, it allows for determination of VA in a wide range of concentrations.

Both procedures, for SD and titration, were verified separately by distillation of acetic acid, acetic acid in the presence of tartaric acid as well as determination of acetic acid, tartaric acid, and total acidity, respectively, with precision (RSD) and accuracy (RE) for both lower than 6.9 and 5.6% respectively. The developed SD and titration systems were applied to determine volatile acidity in samples of dry white and rosé wines separately and as an automated integrated SD–T system. The VA was determined in wine samples and in the same samples spiked with acetic acid. The values of relative error, lower than 6.7%, were obtained for spiked samples. This may be the basis for a conclusion that the method can give accurate results for the determination of volatile acidity in wines. The precision of the obtained results was satisfactory and comparable with the precision of the results obtained using the systems presented in the literature based on pervaporation or gas diffusion [22,25,26,27,28,29]. However, it should be noted that the comparison of precision values is not complete as not all methods presented in the literature report these values [21,23,24].

To sum up, it can be stated that the developed system including automated steam distillation and titration with simple sample handling, and minimal sample pretreatment, has a chance of being a valuable tool for determination of volatile acidity in wine samples.

## 4. Materials and Methods

### 4.1. Instrumentation

The steam distillation module included an aluminum heating block (KSP Elektronika Laboratoryjna, Olsztyn, Poland) and temperature controller Transmit PID G6 (Termipol, Lubliniec, Poland). The flow system consisted of a bidirectional syringe pump SIChrom (FIAlab, Seattle, WA, USA) with a 10-position selection valve (VICI Valco Instruments, Houston, TX, USA) and three syringe pumps (FIAlab, Seattle, WA, USA), each equipped with a nine-position selection valve (FIAlab, Seattle, WA, USA). Cavro glass barrel syringes of a capacity of 1.0 mL (for introducing the sample and titrant) and 2.5 mL (for introducing the nitrogen) were used for the studies. Signals were measured with the use of the USB 4000 Ocean Optics spectrophotometer (Ocean Optics, Dunedin, FL, USA) equipped with fiber optics cables, a halogen light source HL-2000 (Ocean Optics, Dunedin, FL, USA), and an Ultem flow cell with light path length of 10 mm. Measurements were performed at wavelength of 552 nm for determination of acidity in the presence of phenolphthalein, with a reference scan at wavelength of 700 nm. PTFE tubing (0.8 mm i.d.) was used as tubes and as a reaction coil (of 1 mL capacity). PTFE tube (2.0 mm i.d.) surrounded by a thicker flexible tube, through which a stream of cold water was constantly flowing, was used as a cooler. A vessel for the distillate (receiver) was placed on a magnetic stirrer MR 1000 (Heidolph Instruments, Schwabach, Germany). Automatic burette Titronic 300 (SI Analytics, Mainz, Germany) was used to perform batch titration.

### 4.2. Reagents and Solutions

Stock solution of acetic acid (167.83 mmol·L^−1^) was prepared daily by diluting 1 mL acetic acid (96%, d = 1.05 g·L^−1^, POCH, Gliwice, Poland) in water in a 100.0 mL volumetric flask. Stock solution of tartaric acid (344.48 mmol·L^−1^) was prepared by dissolving 5.171 g of C_4_H_6_O_6_ (Merck, Darmstadt, Germany) and making the solution up to 100.0 mL. NaOH solutions were prepared by appropriate dilution of NaOH (0.1000 mol·L^−1^) standard solution (Chempur, Piekary Śląskie, Poland) with water. Phenolphthalein solution (0.2%) was prepared by dissolving 0.2 g of phenolphthalein (POCH, Gliwice, Poland) in 70 mL of 96% ethanol (POCH, Gliwice, Poland) and making the solution up with water to 100 mL. Samples of acetic and tartaric acids were prepared by appropriate dilution of the stock solutions with water. A 1 mL measure of phenolphthalein was added during preparation of samples and NaOH titrant solutions of various concentrations (to NaOH, only for flow-based titration procedure). Nitrogen (Air Products, Warsaw, Poland) was used to form gas segments during flow-based acid–base titration. Reagents of analytical grade were used. Substances for preparation of stock standard solutions were weighed to the nearest 0.0001 g.

Samples of wine were collected at local stores. Samples were degassed for 30 min using an ultrasonic bath (Sonic 3, Warsaw, Poland) and nitrogen was passed through them at the same time to remove gas interferents. Deionized water (0.05 µS·cm^−1^) obtained from the HLP5sp system (Hydrolab, Straszyn, Poland) was used throughout the study. The water was boiled to remove CO_2_.

## 5. Conclusions

The developed approach to determination of volatile acidity in wines, using a system integrating on-line steam distillation and titration, ensures carrying out both processes in a fully mechanized manner. From the analytical point of view, it can obtain results of volatile acidity determination with good precision and accuracy. Sample volume of 6 mL can be subjected to steam distillation. The possibility of using a smaller volume (3 mL), for samples in which the concentration of acetic acid responsible for volatile acidity was expected to be higher, was also confirmed. It was found that using 28 mL of water for steam distillation resulted in transferring all the analyte to the distillate (about 25 mL). Hence, unlike many other methods reported in the literature, the developed procedure of steam distillation does not require calibration. The volatile acidity can be determined in the collected distillate directly in the proposed flow system, using the developed monosegmented flow acid–base titration method. The developed titration procedure does not require calibration and, depending on the titrant concentration, it allows for determination of volatile acidity in a wide range of concentrations. Automated integration of both procedures in a single instrumental system fulfills the requirements of green analytical chemistry and provides a real opportunity to apply it in routine analyses.

## Figures and Tables

**Table 1:** Procedure developed for on-line SD using the system presented in Figure 1; SV—selection valve, SP—syringe pump, SDM—steam distillation module.

Step	SV I Position	SP I Flow Rate (μL s^−1^)	Volume, μL	Action
Washing the system and aspiration of solutions				
1	6	200	4000	Aspiration of water into syringe
2	1	200	4000	Introducing water to SDM
Five repetitions of steps 1–2				
3	2	200	4000	Aspiration of water from SDM into syringe
4	10	200	4000	Transport of water to the waste
Five repetitions of steps 3–4				
5	4	200	4000	Aspiration of sample into syringe
6	10	200	4000	Transport of sample to the waste
7	3	200	4000	Aspiration of acetic acid into syringe
8	10	200	4000	Transport of acetic acid to the waste
9	6	200	4000	Aspiration of water into syringe
10	10	200	4000	Transport of water to the waste
Steam distillation (SD)				
11	6	200	2000	Aspiration of water into syringe
12	1	100	2000	Introducing water to SDM
13	4	100	1000	Aspiration of sample into syringe
14	1	100	3000/6000	Introducing sample to SDM
Waiting 30 s				
15	6	200	4000	Aspiration of water into syringe
16	1	4	4000	Introducing water to SDM
Seven repetitions of steps 15–16				
Washing the system				
17	2	200	4000	Aspiration of solutions from SDM into syringe
18	10	200	4000	Transport of solutions to the waste
Two repetitions of steps 17–18				
19	6	200	4000	Aspiration of water into syringe
20	1	200	4000	Introducing water to SDM
21	2	200	4000	Aspiration of water from SDM into syringe
22	10	200	4000	Transport of water to the waste
Two repetitions of steps 19–22				
23	6	200	4000	Aspiration of water into syringe
24	1	200	4000	Introducing water to SDM
Five repetitions of steps 23–24				
25	2	200	4000	Aspiration of water from SDM into syringe
26	10	200	4000	Transport of water to the waste
Five repetitions of steps 25–26				

**Table 2:** Verification of the on-line SD procedure: results of determination of acetic acid in synthetic samples; NaOH—5.00 mmol·L^−1^, RSD—relative standard deviation (*n* = 3), RE—relative error.

No.	Tartaric Acid (g·L^−1^)	Acetic Acid (g·L^−1^)		RSD (%)	|RE| (%)
		Expected	Determined		
1	-	0.30	0.29	0.7	4.2
2	-	0.45	0.43	4.5	4.3
3	-	0.60	0.59	2.4	1.0
4	-	0.75	0.73	0.6	2.8
5	-	1.20	1.19	6.9	1.1
6	1.72	0.30	0.30	6.2	1.1
7	3.44		0.30	3.8	1.4
8	6.89		0.32	4.0	5.0
9	1.72	0.60	0.59	1.7	1.7
10	3.44		0.61	3.4	1.0
11	6.89		0.60	3.6	0.8

**Table 3:** Verification of the flow-based titration procedure: results of determination of acetic acid (VA) (1–5), tartaric acid (6–11), and total acidity (12–14) in synthetic samples, NaOH—5.00 mmol·L^−1^, RSD—relative standard deviation (*n* = 3), RE—relative error.

No.	Acetic Acid (VA) (1–5)/ Tartaric Acid (6–11)/Total Acidity ^1^ (12–14) (g·L^−1^)		RSD [%]	|RE| [%]
	Expected	Determined		
1	0.15	0.15	0.0	0.0
2	0.30	0.31	5.2	2.8
3	0.45	0.43	3.4	4.0
4	0.60	0.62	3.8	4.0
5	0.75	0.76	0.0	1.2
6	0.09	0.09	4.4	0.5
7	0.17	0.18	3.2	1.6
8	0.22	0.21	3.1	0.4
9	0.43	0.43	5.9	0.5
10	0.86	0.86	4.8	0.2
11	1.72	1.63	0.0	5.6
12	0.18	0.17	3.4	2.0
13	0.26	0.25	0.0	4.8
14	0.53	0.51	3.4	2.9

^1^ Sum of acetic acid and tartaric acid expressed as tartaric acid.

**Table 4:** Results of determination of volatile acidity (VA) in wine samples, RSD—relative standard deviation (*n* = 3); RE—relative error; details in the text.

Sample	Acetic Acid Spiked (g·L^−1^)	VA (g·L^−1^)	RSD (%)	|RE| [%]
White wine (1) ^1^	-	0.83	3.9	-
	0.60	1.40	1.7	5.2
Rosé wine ^1^	-	0.95	9.4	-
	0.90	1.88	3.6	3.3
	1.20	2.07	3.6	6.7
White wine (2) ^2^	-	0.73	3.5	-
	0.60	1.36	7.5	5.0
	1.20	1.98	5.8	4.0

^1^ Traditional titration, using NaOH at a concentration of 5.00 mmol L^−1^. ^2^ Flow-based titration, using NaOH at a concentration of 1.00 and 5.00 (for sample spiked with acetic acid) mmol L^−1^.

## Data Availability

Not applicable.

## Supplementary Materials

The following is available online. Table S1: Titration procedure using the system presented in Figure 1; SV—selection valve, SP—syringe pump.

## Sample Availability

Samples of the compounds are not available from the authors.
