# Supplementary material for: Novel Integrated Flow-Based Steam Distillation and Titration System for Determination of Volatile Acidity in Wines
Source: Molecules. 2021 Dec 18;26(24):7673. doi: 10.3390/molecules26247673 (PMC8708693; doi:10.3390/molecules26247673)
Supplement: Supplementary file 1 [file molecules-26-07673-s001.zip › molecules-1489934-supplementary.pdf]

Novel Integrated Flow-Based Steam Distillation and Titration System for Determination of Volatile Acidity in Wines

Justyna Paluch [1,*], Joanna Kozak [1,*], Karolina Mermer [1], Iwona Molęda [1], Marcin Wieczorek [1], Sławomir Kalinowski [2] and Paweł Kościelniak [1]

[1] Faculty of Chemistry, Jagiellonian University, Gronostajowa 2, 30-387 Krakow, Poland; karolina.mermer@doctoral.uj.edu.pl (K.M.); iwonalo@interia.pl (I.M.); marcin.wieczorek@uj.edu.pl (M.W.); pawel.koscielniak@uj.edu.pl (P.K.)

[2] Department of Chemistry, University of Warmia and Mazury, Plac Łódzki 4, 10-957 Olsztyn, Poland; kalinow@uwm.edu.pl

* Correspondence: justyna.paluch@uj.edu.pl (J.P.); j.kozak@uj.edu.pl (J.K.)

Table S1. Titration procedure using the system presented in Fig. 1; SV – selection valve, SP – syringe pump.

Step	SV position			SP flow rate [mL s^{-1}]			Volume, mL			Action
	II	III	IV	II	III	IV	II	III	IV	
										**Aspiration of solutions**
1	1	-	-	100	-	-	1000	-	-	Aspiration of sample into syringe
2	9	-	-	100	-	-	1000	-	-	Transport sample to the waste
										Two repetitions of steps 1–2
3	1	1	1	100	100	200	1000	1000	2500	Aspiration of sample, titrant and N_2 into syringes
										**Titration**
4	9	-	-	100	-	-	300	-	-	Formation of zone I in mixing coil
5	-	-	9	-	-	100	-	-	200	Introduction of N_2 into mixing coil
6	9	9	-	94	10	-	295	5	-	Formation of zone II in mixing coil
7	-	-	9	-	-	100	-	-	200	Introduction of N_2 into mixing coil
8	9	9	-	94	10	-	290	10	-	Formation of zone III in mixing coil
9	-	-	9	-	-	100	-	-	200	Introduction of N_2 into mixing coil
10	1	1	-	100	100	-	885	15	-	Aspiration of sample and titrant into syringes
11	9	9	-	94	10	-	285	15	-	Formation of zone IV in mixing coil
12	-	-	9	-	-	100	-	-	200	Introduction of N_2 into mixing coil
13	9	9	-	94	10	-	280	20	-	Formation of zone V in mixing coil
14	-	-	9	-	-	100	-	-	200	Introduction of N_2 into mixing coil
15	9	9	-	86	20	-	275	25	-	Formation of zone VI in mixing coil
16	-	-	9	-	-	100	-	-	200	Introduction of N_2 into mixing coil
17	1	1	1	100	100	100	840	60	1200	Aspiration of sample, titrant and N_2 into syringes
18	9	9	-	86	20	-	270	30	-	Formation of zone VII in mixing coil
19	-	-	9	-	-	100	-	-	200	Introduction of N_2 into mixing coil

20	9	9	-	86	20	-	265	35	-	Formation of zone VIII in mixing coil
21	-	-	9	-	-	100	-	-	200	Introduction of N_2 into mixing coil
22	9	9	-	86	20	-	260	40	-	Formation of zone IX in mixing coil
23	-	-	9	-	-	100	-	-	200	Introduction of N_2 into mixing coil
24	1	1	-	100	100	-	795	105	-	Aspiration of sample and titrant into syringes
25	9	9	-	80	30	-	255	45	-	Formation of zone X in mixing coil
26	-	-	9	-	-	100	-	-	200	Introduction of N_2 into mixing coil
27	9	9	-	80	30	-	250	50	-	Formation of zone XI in mixing coil
28	-	-	9	-	-	100	-	-	200	Introduction of N_2 into mixing coil
29	9	9	-	80	30	-	245	55	-	Formation of zone XII in mixing coil
30	-	-	9	-	-	100	-	-	200	Introduction of N_2 into mixing coil
	1	1	1	100	100	100	750	150	1200	Aspiration of sample, titrant and N_2 into syringes
31	9	9	-	80	30	-	240	60	-	Formation of zone XIII in mixing coil
32	-	-	9	-	-	100	-	-	200	Introduction of N_2 into mixing coil
33	9	9	-	74	40	-	235	65	-	Formation of zone XIV in mixing coil
34	-	-	9	-	-	100	-	-	200	Introduction of N_2 into mixing coil
35	9	9	-	74	40	-	235	65	-	Formation of zone XIV in mixing coil
36	-	-	9	-	-	100	-	-	200	Introduction of N_2 into mixing coil
37	9	9	-	74	40	-	230	70	-	Formation of zone XV in mixing coil
38	-	-	9	-	-	100	-	-	200	Introduction of N_2 into mixing coil
39	1	1	-	100	100	-	705	195	-	Aspiration of sample and titrant into syringes
40	9	9	-	74	40	-	225	75	-	Formation of zone XVI in mixing coil
41	-	-	9	-	-	100	-	-	200	Introduction of N_2 into mixing coil
42	9	9	-	74	40	-	220	80	-	Formation of zone XVII in mixing coil
43	-	-	9	-	-	100	-	-	200	Introduction of N_2 into mixing coil
44	9	9	-	67	50	-	215	85	-	Formation of zone XVIII in mixing coil
45	-	-	9	-	-	100	-	-	200	Introduction of N_2 into mixing coil
46	1	1	1	100	100	100	660	240	1200	Aspiration of sample, titrant and N_2 into syringes
47	9	9	-	67	50	-	210	90	-	Formation of zone XIX in mixing coil
48	-	-	9	-	-	100	-	-	200	Introduction of N_2 into mixing coil
49	9	9	-	67	50	-	205	95	-	Formation of zone XX in mixing coil
50	-	-	9	-	-	100	-	-	200	Introduction of N_2 into mixing coil
51	9	9	-	67	50	-	200	100	-	Formation of zone XXI in mixing coil
52	-	-	9	-	-	100	-	-	200	Introduction of N_2 into mixing coil
53	1	1	-	100	100	-	615	285	-	Aspiration of sample and titrant into syringes
54	9	9	-	60	60	-	195	105	-	Formation of zone XXII in mixing coil
55	-	-	9	-	-	100	-	-	200	Introduction of N_2 into mixing coil
56	9	9	-	60	60	-	190	110	-	Formation of zone XXIII in mixing coil

57	-	-	9	-	-	100	-	-	200	Introduction of N_2 into mixing coil
58	9	9	-	60	60	-	185	115	-	Formation of zone XXIV in mixing coil
59	-	-	9	-	-	100	-	-	200	Introduction of N_2 into mixing coil
60	1	1	1	100	100	100	570	330	1200	Aspiration of sample, titrant and N_2 into syringes
61	9	9	-	60	60	-	180	120	-	Formation of zone XXV in mixing coil
62	-	-	9	-	-	100	-	-	200	Introduction of N_2 into mixing coil
63	9	9	-	54	70	-	175	125	-	Formation of zone XXVI in mixing coil
64	-	-	9	-	-	100	-	-	200	Introduction of N_2 into mixing coil
65	9	9	-	54	70	-	170	130	-	Formation of zone XXVII in mixing coil
66	-	-	9	-	-	100	-	-	200	Introduction of N_2 into mixing coil
67	1	1	-	100	100	-	525	375	-	Aspiration of sample and titrant into syringes
68	9	9	-	54	70	-	165	135	-	Formation of zone XXVIII in mixing coil
69	-	-	9	-	-	100	-	-	200	Introduction of N_2 into mixing coil
70	9	9	-	54	70	-	160	140	-	Formation of zone XXIX in mixing coil
71	-	-	9	-	-	100	-	-	200	Introduction of N_2 into mixing coil
72	9	9	-	47	80	-	155	145	-	Formation of zone XXX in mixing coil
73	-	-	9	-	-	100	-	-	200	Introduction of N_2 into mixing coil
74	1	1	1	100	100	100	480	420	1200	Aspiration of sample, titrant and N_2 into syringes
75	9	9	-	47	80	-	150	150	-	Formation of zone XXXI in mixing coil
76	-	-	9	-	-	100	-	-	200	Introduction of N_2 into mixing coil
77	9	9	-	47	80	-	145	155	-	Formation of zone XXXII in mixing coil
78	-	-	9	-	-	100	-	-	200	Introduction of N_2 into mixing coil
79	9	9	-	47	80	-	140	160	-	Formation of zone XXXIII in mixing coil
80	-	-	9	-	-	100	-	-	200	Introduction of N_2 into mixing coil
81	1	1	-	100	100	-	435	465	-	Aspiration of sample and titrant into syringes
82	9	9	-	40	90	-	135	165	-	Formation of zone XXXIV in mixing coil
83	-	-	9	-	-	100	-	-	200	Introduction of N_2 into mixing coil
84	9	9	-	40	90	-	130	170	-	Formation of zone XXXV in mixing coil
85	-	-	9	-	-	100	-	-	200	Introduction of N_2 into mixing coil
86	9	9	-	40	90	-	125	175	-	Formation of zone XXXVI in mixing coil
87	-	-	9	-	-	100	-	-	200	Introduction of N_2 into mixing coil
88	1	1	1	100	100	100	390	510	1200	Aspiration of sample, titrant and N_2 into syringes
89	9	9	-	40	90	-	120	180	-	Formation of zone XXXVII in mixing coil
90	-	-	9	-	-	100	-	-	200	Introduction of N_2 into mixing coil
91	9	9	-	34	100	-	115	185	-	Formation of zone XXXVIII in mixing coil
92	-	-	9	-	-	100	-	-	200	Introduction of N_2 into mixing coil
93	9	9	-	34	100	-	110	190	-	Formation of zone XXXIX in mixing coil
94	-	-	9	-	-	100	-	-	200	Introduction of N_2 into mixing coil

95	1	1	-	100	100	-	345	555	-	Aspiration of sample and titrant into syringes
96	9	9	-	34	100	-	105	195	-	Formation of zone XL in mixing coil
97	-	-	9	-	-	100	-	-	200	Introduction of N_2 into mixing coil
98	9	9	-	34	100	-	100	200	-	Formation of zone XLI in mixing coil
99	-	-	9	-	-	100	-	-	200	Introduction of N_2 into mixing coil
100	9	9	-	27	100	-	95	205	-	Formation of zone XLII in mixing coil
101	-	-	9	-	-	100	-	-	200	Introduction of N_2 into mixing coil
102	1	1	1	100	100	100	300	600	1200	Aspiration of sample, titrant and N_2 into syringes
103	9	9	-	27	100	-	90	210	-	Formation of zone XLIII in mixing coil
104	-	-	9	-	-	100	-	-	200	Introduction of N_2 into mixing coil
105	9	9	-	27	100	-	85	215	-	Formation of zone XLIV in mixing coil
106	-	-	9	-	-	100	-	-	200	Introduction of N_2 into mixing coil
107	9	9	-	27	100	-	80	220	-	Formation of zone XLV in mixing coil
108	-	-	9	-	-	100	-	-	200	Introduction of N_2 into mixing coil
109	1	1	-	100	100	-	255	645	-	Aspiration of sample and titrant into syringes
110	9	9	-	20	100	-	75	225	-	Formation of zone XLVI in mixing coil
111	-	-	9	-	-	100	-	-	200	Introduction of N_2 into mixing coil
112	9	9	-	20	100	-	70	230	-	Formation of zone XLVII in mixing coil
113	-	-	9	-	-	100	-	-	200	Introduction of N_2 into mixing coil
114	9	9	-	20	100	-	65	235	-	Formation of zone XLVIII in mixing coil
115	-	-	9	-	-	100	-	-	200	Introduction of N_2 into mixing coil
116	1	1	1	100	100	100	210	690	1200	Aspiration of sample, titrant and N_2 into syringes
117	9	9	-	20	100	-	60	240	-	Formation of zone XLIX in mixing coil
118	-	-	9	-	-	100	-	-	200	Introduction of N_2 into mixing coil
119	9	9	-	14	100	-	55	245	-	Formation of zone L in mixing coil
120	-	-	9	-	-	100	-	-	200	Introduction of N_2 into mixing coil
121	9	9	-	14	100	-	50	250	-	Formation of zone LI in mixing coil
122	-	-	9	-	-	100	-	-	200	Introduction of N_2 into mixing coil
123	1	1	-	100	100	-	255	645	-	Aspiration of sample and titrant into syringes
124	9	9	-	14	100	-	45	255	-	Formation of zone LII in mixing coil
125	-	-	9	-	-	100	-	-	200	Introduction of N_2 into mixing coil
126	9	9	-	14	100	-	40	260	-	Formation of zone LIII in mixing coil
127	-	-	9	-	-	100	-	-	200	Introduction of N_2 into mixing coil
128	9	9	-	7	100	-	35	265	-	Formation of zone LIV in mixing coil
129	-	-	9	-	-	100	-	-	200	Introduction of N_2 into mixing coil
130	1	1	1	100	100	100	120	780	1200	Aspiration of sample, titrant and N_2 into syringes
131	9	9	-	7	100	-	30	265	-	Formation of zone LV in mixing coil
132	-	-	9	-	-	100	-	-	200	Introduction of N_2 into mixing coil

133	9	9	-	7	100	-	25	275	-	Formation of zone LVI in mixing coil
134	-	-	9	-	-	100	-	-	200	Introduction of N_2 into mixing coil
135	9	9	-	7	100	-	20	280	-	Formation of zone LVII in mixing coil
136	-	-	9	-	-	100	-	-	200	Introduction of N_2 into mixing coil
137	1	1	-	100	100	-	75	825	-	Aspiration of sample and titrant into syringes
138	9	9	-	4	100	-	15	285	-	Formation of zone LVIII in mixing coil
139	-	-	9	-	-	100	-	-	200	Introduction of N_2 into mixing coil
140	9	9	-	4	100	-	10	290	-	Formation of zone LIX in mixing coil
141	-	-	9	-	-	100	-	-	200	Introduction of N_2 into mixing coil
142	9	9	-	4	100	-	5	295	-	Formation of zone LX in mixing coil
143	-	-	9	-	-	100	-	-	200	Introduction of N_2 into mixing coil
144	1	1	-	100	100	-	30	870	-	Aspiration of sample and titrant into syringes
145	-	9	-	-	100	-	0	300	-	Formation of zone LXI in mixing coil
146	-	-	9	-	-	100	-	-	200	Introduction of N_2 into mixing coil
